# Renal Expression and Urinary Excretion of Na-K-2Cl Cotransporter in Obstructive Nephropathy

**DOI:** 10.1155/2017/7171928

**Published:** 2017-01-10

**Authors:** Anabel Brandoni, Adriana M. Torres

**Affiliations:** Farmacología, Facultad de Ciencias Bioquímicas y Farmacéuticas, Universidad Nacional de Rosario, CONICET, Santa Fe, Argentina

## Abstract

Renal damage due to urinary tract obstruction accounts for up to 30% of acute kidney injury in paediatrics and adults. Bilateral ureteral obstruction (BUO) is associated with polyuria and reduced urinary concentrating capacity. We investigated the renal handling of water and electrolytes together with the renal expression and the urinary excretion of the Na-K-Cl cotransporter (NKCC2) after 1 (BUO-1), 2 (BUO-2), and 7 (BUO-7) days of release of BUO. Immunoblotting and immunohistochemical studies showed that NKCC2 expression was upregulated in apical membranes both from BUO-2 and from BUO-7 rats. The apical membrane expression, where NKCC2 is functional, may be sufficient to normalize water, potassium, sodium, and osmolytes tubular handling. NKCC2 abundance in homogenates and mRNA levels of NKCC2 was significantly decreased in almost all groups suggesting a decrease in the synthesis of the transporter. Urinary excretion of NKCC2 was increased in BUO-7 groups. These data suggest that the upregulation in the expression of NKCC2 in apical membranes during the postobstructive phase of BUO could contribute to improving the excretion of sodium and consequently also the excretion of potassium, osmolytes, and water. Moreover, the increase in urinary excretion of NKCC2 in BUO-7 group could be a potential additional biomarker of renal function recovery.

## 1. Introduction

Renal damage due to urinary tract obstruction accounts for up to 30% of acute kidney injury in paediatrics [1]. Urinary tract obstruction is characterized by a significantly reduced capacity of kidney to regulate urinary excretion of water and sodium [2–4]. The loss of urinary concentrating ability involves all nephron segments and the underlying pathophysiology is intricate [4]. The active transport of sodium chloride in the thick ascending limb of Henle's loop occurs mainly through the Na-K-2Cl cotransporter type 2 (NKCC2). Thus, corticomedullary concentration gradient is created. This interstitial hypertonicity in the renal medulla leads to water absorption through the aquaporin-2 (AQP2) water channel in the collecting duct in the presence of vasopressin [5, 6].

Many studies in animal models have shown that alteration in sodium transporters plays important roles in disorders of sodium and water balance, including lithium-induced nephrogenic diabetes insipidus [7], vitamin D-induced hypercalcemia [8], and liver cirrhosis [9]. The pathophysiology of urine concentrating ability after release of bilateral ureteral obstruction (BUO) is complicated and the molecular mechanisms involved are not yet fully clarified.

Sodium transporter proteins, such as Na^+^/H^+^ exchanger type 3 (NHE3), NKCC2, and thiazide-sensitive Na^+^-Cl^−^ cotransporter, are excreted in urine as previous studies have demonstrated [10]. However, the presence in urine of integral membrane proteins, such as NKCC2 in BUO rats, has not yet been elucidated and it seems likely to be important in clinical trials.

The aim of this study was to examine the time course of renal NKCC2 expression after the release of BUO employing Western blotting (in homogenates and apical membranes), immunohistochemistry, and RT-PCR techniques. In addition, the influence of NKCC2 expression on renal function parameters and urinary excretion of NKCC2 after release of ureteral occlusion was also evaluated.

## 2. Materials and Methods

### 2.1. Ethical Approval

Male Wistar rats 110 to 130 days old were used throughout the study. Animals were allowed free access to a standard laboratory chow and tap water and were housed in an environment of constant temperature and humidity with regular light cycles (12 h) during the experiments. Animals were cared for in accordance with principles and guidelines for the care and use of laboratory animals, recommended by the National Academy of Sciences and published by the National Institute of Health (NIH publication 7th edition revised 1996) and recommended by regulations of the local ethics committee. All experimental procedures were approved by the Faculty of Biochemical and Pharmaceutical Sciences Institutional Animal Care and Use Committee.

### 2.2. Experimental Animals

Ureters' ligation and release were performed as previously described [11–14]. The abdominal cavity was opened, and a nontraumatic microvascular clamp was placed on both proximal ureters. After closure of the abdomen, the animals were kept for 24 hours while they were given food and water ad libitum. The ureteral obstruction was released after 24 h and the rats, referred to as bilateral ureteral obstructed ones, were kept alive for different times. Corresponding control groups, referred to as sham group, were treated the same, except that no ureteral obstruction was performed. Rats were anaesthetized with sodium thiopental (70 mg/kg b.w., i.p.).

The following groups were studied:

(i) BUO-1 (*n* = 5); experimental studies were performed at 1 day after ureteral release.
(ii) BUO-2 (*n* = 6): experimental studies were performed at 2 days after ureteral release.
(iii) BUO-7 (*n* = 5): experimental studies were performed at 7 days after ureteral release.

As all the tested parameters in sham groups (1, 2, and 7 days after sham surgery) were similar, we decided to consider them as one group (unified sham) to facilitate the analysis of the results.

Different sets of experimental animals were used for biochemical determinations, renal clearance studies, preparation of homogenates (H), and apical membranes (AM) for Western blotting studies, immunohistochemical studies, and mRNA isolation (for RT-PCR studies).

### 2.3. Biochemical Determinations

On the day of the experiment, blood was withdrawn from femoral artery and urine was collected by bladder puncture as previously described [12–14]. Serum samples were used to measure urea and creatinine levels as indicative parameters of global renal function. Urine samples were centrifuged at 1000 ×g for 10 minutes to discard whole cells and detritus and the supernatant were used for evaluating creatinine concentration and NKCC2 abundance. Serum urea and creatinine levels, as well as urine creatinine concentrations, were determined employing commercial kits (Wiener Laboratory, Rosario, Argentina).

### 2.4. Renal Clearance Studies

These studies were performed as previously described [13–15]. The glomerular filtration rate (GFR) was calculated from the clearance of inulin. The ratio urine to plasma osmolality (U/P), the fractional excretion of water (FE% H_2_O), sodium (FE% Na), potassium (FE% K), and osmolytes (FE% Osm) were also calculated by conventional formulae for each animal. Inulin concentrations in plasma and urine were determined as previously described [12–15]. Sodium and potassium were measured by flame photometry and the volume of urine by gravimetry. Osmolalities were determined in a freezing point osmometer (Osmomat 030, Berlin, Germany).

### 2.5. Preparation of Apical Membranes (AM)

All rats were anaesthetized as previously described and kidneys were rapidly removed. The renal tissue was cleaned, dried, weighed, and placed in saline. The kidney samples were dissected in cortex and medulla. Medulla samples included outer and inner medulla. AM were isolated from renal cortex and medulla by Mg/EDTA precipitation as previously described [16]. The AM pellets obtained were resuspended in 50 mM mannitol plus 10 mM Tris-HEPES buffer (pH 7.40), and total protein concentrations in these membrane fractions were measured as previously described [12–14]. Each preparation represents cortical and medullary tissue from four animals. Four preparations were obtained for each experimental group.

### 2.6. Electrophoresis and Immunoblotting

Homogenate (50 *μ*g proteins), AM (40 *μ*g proteins), and urine samples (10 *μ*L) were boiled for 3 min in the presence of 1% 2-mercaptoethanol/2% SDS (sodium dodecyl sulphate). Samples were applied to a 5% polyacrylamide gel, separated by SDS-PAGE, and then electroblotted to nitrocellulose membranes as previously described [12–18]. To verify equal protein loading and transfer between lanes, Ponceau Red and antibody against human *β*-actin were used as previously reported [12–18]. The nitrocellulose membranes were incubated 1 h with 5% nonfat dry milk in phosphate buffer saline containing 0.1% Tween 20 (PBST). After being rinsed with PBST, the membranes were then incubated overnight at 4°C with a commercial polyclonal antibody against rat NKCC2 (NKCC21-A; 1.25 *μ*g/mL; Alpha Diagnostic International, San Antonio, USA) or a commercial mouse monoclonal antibody against human *β*-actin (at a dilution of 1 : 800; Alpha Diagnostic International, San Antonio, TX, USA). Blots were processed for detection using a commercial kit (ECL Enhanced Chemiluminescence System, Thermo Scientific, Rockford, USA). The immunoreactive bands were quantified using the Gel-Pro Analyzer (Media Cybernetics, Silver Spring, USA) software in all Western blotting studies. For densitometry of immunoblots, samples from obstructed kidneys were run on each gel with corresponding sham kidneys. NKCC2 abundance was normalized to *β*-actin. The relative protein expression for NKCC2 was expressed as percentage of the mean sham value for each gel.

### 2.7. RNA Isolation and RT-PCR

Total RNA was isolated using the TriZOL® reagent (Invitrogen, Carlsbad, USA) according to the manufacturer's instructions. RNA was dissolved in DEPC-treated water and kept at −80°C until use. Reverse transcription of 5 *μ*g of total RNA was performed with oligo-dT primer, and cDNA samples were stored at −20°C until assayed. NKCC2 and 18S, as housekeeping gene, cDNAs were amplified with specific antisense primers that shared the same sense primer. Sequences were as follows: 5′-ACCAAGAACCTCCCTCCTGT-3′ (NKCC2 sense), 5′-TCGGACACCAAGGTACAACA-3′ (NKCC2 antisense) and 5′-CGCGGTTCTATTTTGTTGGT-3′ (18S sense), 5′-AGTCGGCATCGTTTATGGTC-3′ (18S antisense). The thermoprofile for SYBR RT-PCR was 94°C for 2 min for denaturation, followed by 23 cycles for both NKCC2 and 18S, 94°C for 15 s, and 55°C for 30 s and 1 min. RT-PCR products were then resolved on a 2% agarose gel stained with Sybr Safe™ and bands were visualized using a high performance ultraviolet transilluminator (Safe Imager™, Invitrogen, Carlsbad, USA). Images of the RT-PCR Syber Safe agarose gels were acquired and quantification of the optical density (OD) of bands was performed using the Gel-Pro Analyzer (Media Cybernetics, Silver Spring, USA) software. Results were expressed as the ratio between the intensities of NKCC2 OD and 18S OD.

### 2.8. Immunohistochemistry

As previously described [13–18], rats were briefly perfused with saline, followed by perfusion with periodate-lysine-paraformaldehyde solution (0.01 M NaIO_4_, 0.075 M lysine, 0.0375 M phosphate buffer, with 2% paraformaldehyde, pH: 6.20), through a cannula inserted in the abdominal aorta. Kidney slices were immersed in periodate-lysine-paraformaldehyde solution at 4°C overnight. The tissue was embedded in paraffin. Paraffin sections were cut. After deparaffining, the sections were incubated with 3% H_2_O_2_ for 15 min (to eliminate endogenous peroxidase activity) and then with blocking serum for 30 min. The sections were then incubated with noncommercial polyclonal antibody against NKCC2 (dilution 1 : 1000) overnight at 4°C kindly provided by Dr. Mitsunobu Matsubara (Division of Molecular Medicine, Centre for Translational and Advanced Animal Research, Tohoku University School of Medicine, Aoba-ku, Sendai, Japan) [19]. The sections were rinsed with Tris-buffer saline containing 0.1% Tween 20 (TBST). The sections were incubated with horseradish peroxidase- (HRP-) conjugated secondary antibody against rabbit immunoglobulin for 1 h. To detect HRP labeling, a commercial kit was used (DAB Substrate Kit, Zymed Laboratories Inc., San Francisco, USA). The sections were counterstained with hematoxylin before being examined under a light microscope. Two blinded investigators independently examined the respective samples.

### 2.9. Materials

Chemicals were purchased from Sigma (St. Louis, USA) and were of analytical pure grade. Urea and creatinine kits were from Wiener Lab, Argentina. The nontraumatic microvascular clamps (Schwartz clamp strong ang, ref 26-1043) were manufactured by Miltex Instrument Company, Inc., Bethpage, NY, USA, and were purchased from Thomas Scientific, NJ, USA.

### 2.10. Statistical Analysis

All results are expressed as mean ± standard error (SE). Statistical differences between groups were evaluated by one-way ANOVA followed by the Newman-Keuls test. *P* < 0.05 was considered statistically significant. For these analyses GraphPad (San Diego, USA) software was used.

## 3. Results

Table 1 shows urea and creatinine concentration in plasma, the urine to plasma osmolality ratio (U/P), and the urine volume in all experimental groups. In BUO-1 a highly significant increase in both urea and creatinine plasma levels indicated the presence of acute renal failure in our experimental model. BUO-2 group showed a partial recovery of these parameters but still remained above normal values thus confirming acute renal failure in these animals too. Early time after release of BUO (BUO-1 and BUO-2) resulted in an increase in urine output and parallel reduction in urine osmolality and U/P, indicating an impairment of urinary concentrating capacity. At 7 days after release of BUO, full recovery to normal values was found in all of these parameters.

BUO release was also associated with altered renal electrolytes and water management. One day after the release of BUO, there were significant increases in the fractional excretion of water (FE% H_2_O), potassium (FE% K), and osmolytes (FE% Osm). Two days after the release of BUO all of these parameters persisted elevated and returned to normal levels after 7 days of releasing the obstruction (Table 2). On the other hand, the fractional excretion of sodium (FE% Na) was recovered to control levels at day 2 of releasing. However, a statistical comparison between BUO-2 and sham groups was also performed by standard unpaired* t*-test, which showed that there was a significant difference between them (Table 2).

In the present study, we used semiquantitative immunoblotting, immunohistochemistry, and RT-PCR to investigate expression changes of renal NKCC2 for a period of time after release of bilateral ureteral obstruction.

**Figure 1:**
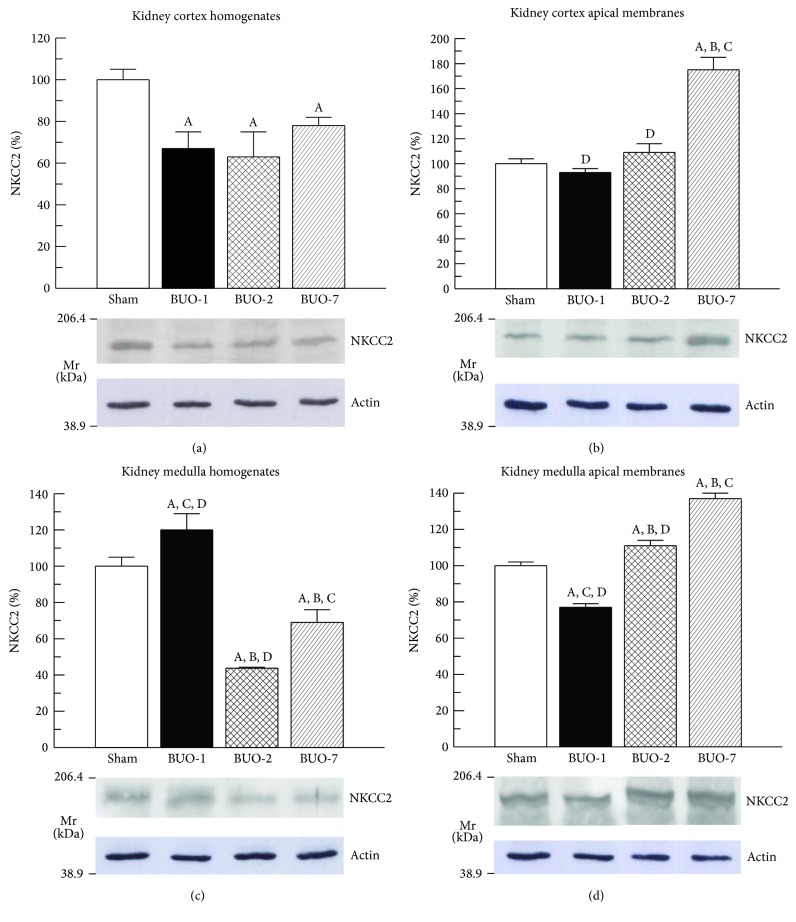
Renal cortex homogenates ((a) 40 *μ*g proteins) and apical membranes ((b) 30 *μ*g proteins) and renal medullae homogenates ((c) 40 *μ*g proteins) and apical membranes ((d) 30 *μ*g proteins) from kidneys of sham and BUO rats were separated by sodium dodecyl sulphate-polyacrylamide gel electrophoresis (5%) and blotted onto nitrocellulose membranes. NKCC2 (150 kDa) was identified using commercial polyclonal antibodies as described in Section 2. Kaleidoscope-prestained standards of molecular mass (Mr) corresponding to myosin (206.4 kDa) and to carbonic anhydrase (38.9 kDa) are indicated in the left of the figure. Sham levels were set at 100%. Each column represents mean ± SE from experiments carried out in triplicate on four different cortex and medulla homogenates and apical membranes preparations for each experimental group. ^A^*P* < 0.05 versus sham, ^B^*P* < 0.05 versus BUO-1, ^C^*P* < 0.05 versus BUO-2, and ^D^*P* < 0.05 versus BUO-7.

Western blotting in homogenates and AM from kidney cortex and medulla from sham-operated and BUO rats showed signals for NKCC2 (Figure 1). The expression of NKCC2 in cortex homogenate was reduced after 1-day release of BUO and persisted downregulated up to 7 days after release. NKCC2 expression level in AM of kidney cortex was not different between both BUO-1 and BUO-2 compared with sham-operated animals. This parameter in BUO-7 rats, in contrast, was strongly increased.  Figure 1(c) shows increased NKCC2 expression in kidney medulla homogenates from BUO-1 rats. Two days after releasing the obstruction there was an important decrease in this parameter that tended to return to normal levels at 7 days after release of BUO. In Figure 1(d), it is possible to observe that NKCC2 expression level in kidney medulla of AM was significantly decreased in BUO-1 animals but increased in both BUO-2 and BUO-7 rats.

**Figure 2:**
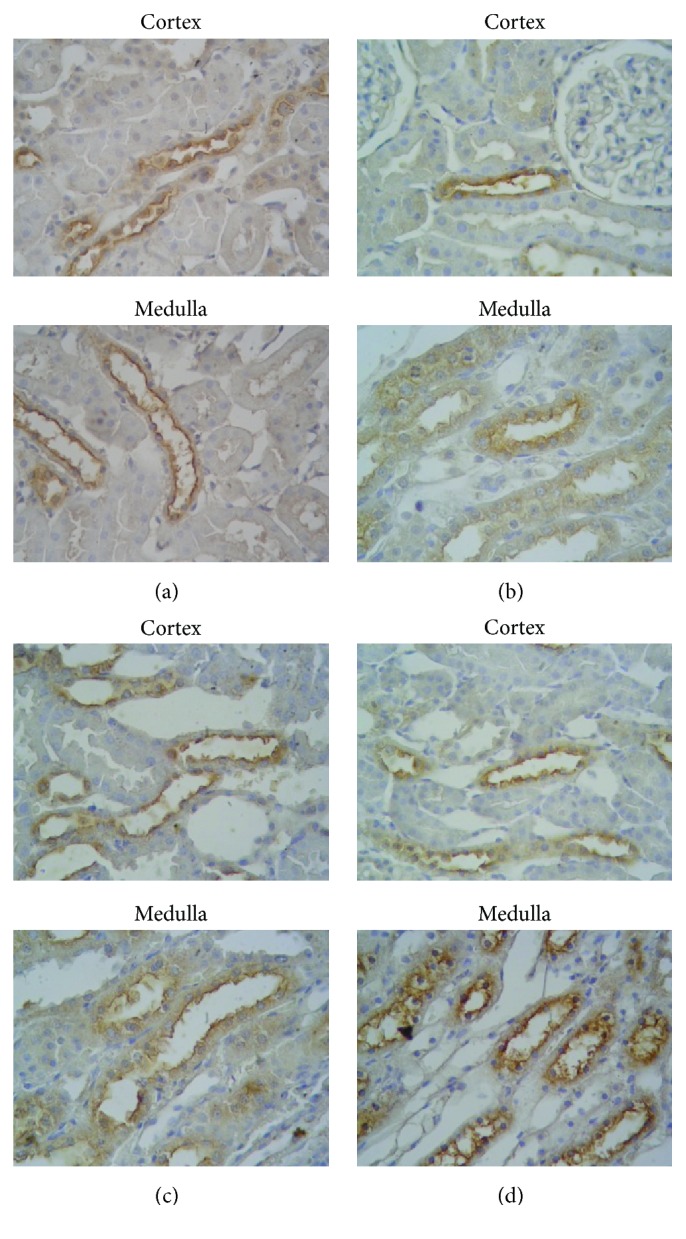
Immunohistochemistry for NKCC2 in cortex and medulla kidney of sham (a) and BUO (b–d) rats. Serial sections from each rat kidney were stained with noncommercial polyclonal anti-NKCC2 antibodies as described in Section 2. Immunoperoxidase microscopy demonstrated association of NKCC2 labeling with apical membrane of thick ascending limb of Henle's loop of sham rats (a). No difference in NKCC2 labeling was found in cortex of BUO-1 (b) and BUO-2 (c); increased labeling was seen in apical membrane in BUO-7 (d) kidneys. NKCC2 labeling in renal medullae was reduced in BUO-1 (b) and increased apical localization of NKCC2 in BUO-2 and BUO-7 (c-d) was observed. These figures are representatives of typical samples from four rats. Magnification ×400.

We have also examined NKCC2 expression in sham and BUO rats using immunohistochemistry (Figure 2). NKCC2 was decreased in BUO-1 in cortex and medulla (Figure 2(b)). Immunohistochemical observations revealed a predominant apical NKCC2 localization, two and seven days after release of the obstruction (Figures 2(c) and 2(d)); these results confirmed the pattern observed for immunoblotting.

**Figure 3:**
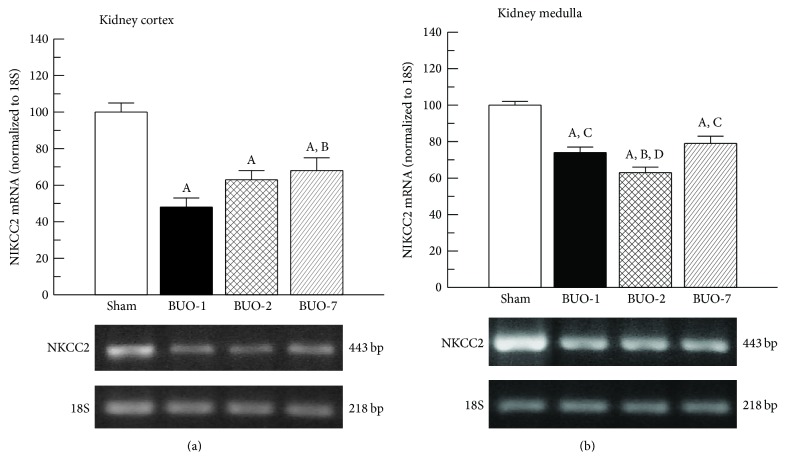
mRNA levels of NKCC2 cotransporter in cortex (a) and medulla (b) of sham and BUO rats. Total RNA was isolated and NKCC2 and 18S mRNA were assessed by RT-PCR analysis. Relative mRNA levels were quantitated, and NKCC2 levels were normalized to 18S mRNA. Ratios are represented in graphical form, and the data are representative of four experiments. ^A^*P* < 0.05 versus sham, ^B^*P* < 0.05 versus BUO-1, ^C^*P* < 0.05 versus BUO-2, and ^D^*P* < 0.05 versus BUO-7.

To investigate the molecular mechanism of the downregulation of NKCC2 protein expression in renal medullae and cortex, NKCC2 mRNA was quantified by RT-PCR in BUO and sham animals (Figure 3). Interestingly, NKCC2 mRNA was significantly decreased in the medulla and cortex of all BUO rats.

**Figure 4:**
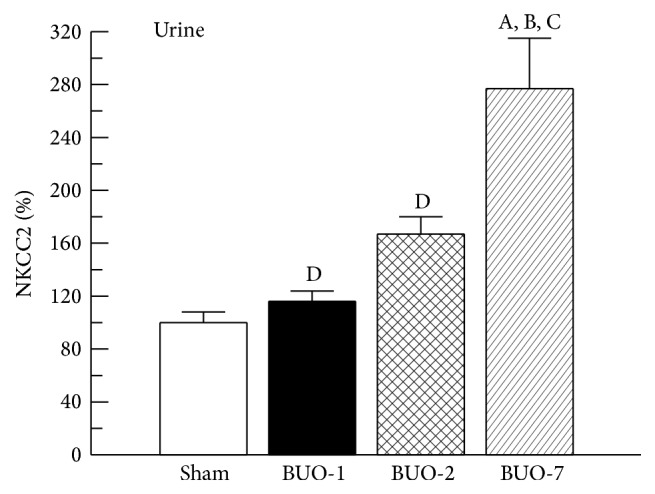
Urinary excretion of NKCC2 in sham and BUO rats. Urine samples were separated by SDS-PAGE (8.5%) and blotted onto nitrocellulose membranes. NKCC2 was identified using commercial polyclonal antibody as described in Section 2. The densitometric quantification of urine NKCC2 immunoblotting is expressed as the percentage of a ratio of arbitrary units relative to urinary creatinine concentration for each sample. Sham levels were set at 100%. Results are expressed as mean values ± SE. ^A^*P* < 0.05 versus sham, ^B^*P* < 0.05 versus BUO-1, ^C^*P* < 0.05 versus BUO-2, and ^D^*P* < 0.05 versus BUO-7.

NKCC2 abundance in urine was related to urinary creatinine concentrations to correct variations in urine output as previously described for other urinary transporters and enzymes [16–18, 20–22]. Measurements of biomarker abundance themselves are insufficient because normal physiological variations in water excretion may dilute or concentrate urinary proteins. As the creatinine is excreted in the urine at relatively constant rate, it is used to normalize the urinary excretion of a particular protein. Urinary excretion of NKCC2 was almost three times higher in BUO-7 group compared with other groups studied, as shown in Figure 4.

## 4. Discussion

The kidney disease caused by impaired flow of urine or tubular fluid is known as obstructive nephropathy. It is a common disorder that occurs at all ages [2, 4]. Up to 30% of acute kidney injury in pediatrics may be explained by kidney injury due to urinary tract obstruction, especially in nephrolithiasis [1, 23].

In experimental models of ureteral obstruction, in several species, it has been verified that natriuresis and vasopressin-resistant polyuria appeared after ureteral obstruction in patients [2, 24]. Moreover, in vitro studies have demonstrated a decrease in reabsorption of water and salt in the proximal tubule, the distal convoluted tubule, and the collecting duct after release of obstruction [25, 26]. In the present study we have evaluated the changes in renal expression and in urinary excretion of NKCC2 during the postobstructive phase of bilateral ureteral obstruction. Emphasis was placed on the changes during the postobstructive phase of the release of bilateral ureteral occlusion in the expression of renal NKCC2 assayed by Western blotting and immunohistochemistry, its relationship with NKCC2 urinary excretion and with renal functional changes.

Apical NKCC2 and basolateral Na,K-ATPase are major sodium transporters responsible for active NaCl transport in the thick ascending limb of Henle's loop (mTAL). In particular, NKCC2 cotransporter in the mTAL is partly responsible for the generation of a high osmolality in the interstitium by performing countercurrent multiplier, which is dependent on NaCl reabsorption [4].

The abundance of major renal sodium transporters/cotransporters and transporters of urea was reduced along the nephron in response to unilateral ureteral obstruction (UUO) [27, 28]; however, this model with one unobstructed hyperfunctioning kidney is not directly comparable to the present model of BUO. BUO and release of BUO in rats were associated with decreased expression of the major renal sodium transporters along the nephron and collecting ducts. In fact, early studies showed that expression of NKCC2 in samples of whole kidney was drastically reduced at day 3 after release of BUO [29]. In this regard, we observed in this study a decrease in the expression of NKCC2 both in cortex and in medulla homogenates at day 2 after release of BUO, which might precede the dramatic decrease at day 3 after release of BUO described by Li et al. [29]. It is important to mention that our present work emphasizes the NKCC2 expression in the apical membrane where the protein is functional.

The present study demonstrated that after release of ureteral obstruction follows a marked diuresis characterized by massive loss of water, sodium, and other solutes. In this regard, NKCC2 expression was significantly downregulated in cortical and medullary homogenates in almost all groups except for renal medulla from BUO-1 group. Increased abundance of NKCC2 in medullary homogenates of this group could be caused by a decrease in its degradation with the aim of reaching a compensation to the sudden decrease of this transporter in renal cortex. This downregulation of NKCC2 expression would cause a profound increase in the urinary excretion of sodium and chloride (i.e., up to 25% of the filtered load of Na). The consequent alteration of the transepithelial potential difference in the thick ascending limb will also result in a significant increase in the excretion of calcium and magnesium. The increased delivery of sodium to the distal tubule will augment the depolarization of the luminal membrane of this nephron segment which will facilitates potassium urinary excretion. The reduced sodium chloride reabsorption in the thick ascending limb due to the decrease of NKCC2 expression will also interfere with the critical step in the mechanism that produces a hypertonic medullary interstitium, causing an increase in the elimination of water in urine [4].

In cortical apical membrane, NKCC2 expression increased in BUO-7 group. NKCC2 expression also increased at 2 and 7 days after release of BUO in apical membranes of medulla. These results could explain, at least in part, the recovery of the fractional excretion of sodium, and consequently potassium, osmolytes, and water observed in BUO-7 group.

The downregulation of the expression of NKCC2 protein in homogenates from BUO kidneys could be caused by the decrease in its synthesis, the increase in its degradation, the liberation of intracellular and apical membrane fractions into tubular fluid, or a consequence of cell shedding. Moreover, increased NKCC2 abundance in the apical membrane together with previous results in homogenates suggests alterations in the intracellular distribution of this transporter. An alteration in NKCC2 trafficking might be caused by an increased recruitment of preformed transporters into the membranes or an inhibition in the internalization of membrane transporters.

Furthermore, immunohistochemical study confirms that there was an increase in NKCC2 expression at apical plasma membrane domains of thick ascending limb cells of BUO compared to sham-operated rats.

We have also observed that the level of NKCC2 mRNA as well as protein abundance in homogenate in cortex and medulla decreased after release of BUO, indicating a downregulation at transcriptional level. However, other mechanisms mentioned above cannot be excluded.

Measurements of renal excretion of different carrier proteins are exploited for the study of certain disease states [16, 22, 30, 31]. It has been demonstrated that Na^+^ transporter proteins such as NKCC2 are excreted in the urine of normal rats in detectable quantities [10]. NKCC2 excretion in urine of sham and BUO rats was determined. The initial low-speed centrifugation was performed to remove whole cells, so it is unlikely that the appearance of NKCC2 cotransporter in urine is due to sloughing of whole cells. The presence of this cotransporter in urine may be exploitable in clinical studies as a potential marker of recovery after release of BUO because we found an important increase in NKCC2 urinary excretion in BUO-7 group. It could also help understand the whole scene about the impaired ability to concentrate urine during postobstructive phase of bilateral ureteral occlusion.

## 5. Conclusion

Data from this study suggest that the upregulation in NKCC2 expression in apical membranes during the postobstructive phase of bilateral ureteral occlusion could improve the excretion of sodium and consequently the excretion of potassium, osmolytes, and water. Moreover, the increase in urinary excretion of NKCC2 in BUO-7 group could be a potential additional biomarker of renal function recovery, suitable to complete the panel of biomarkers currently compiled to evaluate the onset and recovery of obstructive nephropathy.

## Figures and Tables

**Table 1:** Creatinine and urea plasma levels, urine volume, and urine to plasma osmolality ratio (U/P) in Sham, BUO-1, BUO-2, and BUO-7 rats.

	Sham (*n* = 16)	BUO-1 (*n* = 5)	BUO-2 (*n* = 6)	BUO-7 (*n* = 5)
Plasma creatinine (mg/dL)	0.35 ± 0.03	1.40 ± 0.20^a,c,d^	0.97 ± 0.12^a,b,d^	0.51 ± 0.06^b,c^
Plasma urea (g/L)	0.56 ± 0.09	2.91 ± 0.67^a,c,d^	1.78 ± 0.61^a,b,d^	0.60 ± 0.09^b,c^
Urine volume (*μ*L/min/100 g b.w)	5.01 ± 0.74	11.60 ± 1.12^a,c,d^	7.95 ± 1.17^b^	5.23 ± 0.52^b^
U/P	4.19 ± 0.41	1.83 ± 0.21^a,d^	2.10 ± 0.17^a,d^	3.79 ± 0.25^b,c^

Results are expressed as means ± SE. BUO: bilateral ureteral obstruction; BUO-1: 1 day after releasing of BUO; BUO-2: 2 days after releasing of BUO; BUO-7: 7 days after releasing of BUO; sham: sham-operated rats. Statistical comparison between experimental groups was made by one-way ANOVA plus Newman-Keuls. ^a^*P* < 0.05 versus sham, ^b^*P* < 0.05 versus BUO-1, ^c^*P* < 0.05 versus BUO-2, and ^d^*P* < 0.05 versus BUO-7.

**Table 2:** Changes in the fractional excretion of H_2_O (EF% H_2_O), K (EF% K), Osm (EF% Osm), and Na (EF% Na) in Sham, BUO-1, BUO-2, and BUO-7 rats.

	Sham (*n* = 16)	BUO-1 (*n* = 5)	BUO-2 (*n* = 6)	BUO-7 (*n* = 5)
EF% H_2_O	0.86 ± 0.12	9.86 ± 1.57^a,c,d^	5.04 ± 0.93^a,b,d^	0.89 ± 0.10^b,c^
EF% K	30.5 ± 2.5	87.6 ± 5.5^a,d^	78.2 ± 8.2^a,d^	39.4 ± 3.1^b,c^
EF% Osm	2.88 ± 0.12	15.14 ± 2.01^a,c,d^	9.81 ± 0.82^a,b,d^	3.29 ± 0.28^b,c^
EF% Na	1.28 ± 0.09	50.3 ± 11.6^*∗*,a,c,d^	2.96 ± 0.15^*∗*,b^	1.46 ± 0.08^b^

Values are presented as means ± SE. BUO: bilateral ureteral obstruction; BUO-1: 1 day after releasing of BUO; BUO-2: 2 days after releasing of BUO; BUO-7: 7 days after releasing of BUO; Sham: sham-operated rats. Statistical comparison between experimental groups was made by a standard unpaired *t*-test ^*∗*^*P* < 0.05 versus Sham; or one-way ANOVA plus Newman-Keuls ^a^*P* < 0.05 versus Sham, ^b^*P* < 0.05 versus BUO-1, ^c^*P* < 0.05 versus BUO-2, and ^d^*P* < 0.05 versus BUO-7.
